# OmicsPred as a centralised resource for genetic prediction of multi-omic traits

**DOI:** 10.64898/2026.05.15.26353298

**Published:** 2026-05-19

**Authors:** Carles Foguet, Laurent Gil, Yu Xu, Sofía Salazar-Magaña, Scott C. Ritchie, Elodie Persyn, Hae Kyung Im, Michael Inouye, Samuel A. Lambert

## Abstract

Genetic prediction of multi-omic data has emerged as a cost-effective alternative to direct omics profiling, particularly useful for identifying molecular features associated with disease susceptibility. However, despite its popularity, multi-omic imputation models are fragmented across studies, hindering findability, accessibility, interoperability and re-use. To address this, we developed OmicsPred ( https://www.omicspred.org ), a centralised platform for the deposition and dissemination of genetic prediction models of multi-omic traits. OmicsPred unifies the most commonly used molecular imputation models (e.g. from PredictDB) and other published studies totalling 3,339,469 prediction models spanning transcriptomic, proteomic, and metabolomic traits (as of May 2026). Each model is accompanied by metadata describing score development and predictive performance, and distributed in formats compatible with popular analytic tools, such as PGS Catalog Calculator and MetaXcan. To demonstrate the utility of the resource for systematic target discovery, we perform a multi-omic phenome-wide association analysis in Million Veterans Program data.

## Full Text Availability

The license terms selected by the author(s) for this preprint version do not permit archiving in PMC. The full text is available from the preprint server.

