# Supplementary material for: OmicsPred as a centralised resource for genetic prediction of multi-omic traits
Source: medRxiv. 2026 Jun 11:2026.05.15.26353298. Originally published 2026 May 19. Preprint. [Version 2] doi: 10.64898/2026.05.15.26353298 (PMC13228761; doi:10.64898/2026.05.15.26353298)
Supplement: Supplementary file 1 [file media-1.pdf]

Supplemental Material

Supplemental Notes **1**
1. OmicsPred scoring file and metadata description 1
Performance Metrics (used to describe training and testing performance for scores) 6
2. OmicsPred data access and implementation 8
3. Inclusion Criteria for OmicsPred 8
Supplemental Tables **9**
Supplemental Figures **11**
Supplemental References **16**

Supplemental Notes

1. OmicsPred scoring file and metadata description

The goal of the OmicsPred resource is to make the genetic scores used for molecular imputation and association studies findable, accessible, interoperable and re-usable in accordance with FAIR Data Principles[1].

The OmicsPred scoring file format is based on the scoring file format of the PGS Catalog[2] (https://www.pgscatalog.org/downloads/#dl_ftp_scoring). Each scoring file is labelled by its OPGS ID (e.g. OPGS000001.txt). The scoring files are zipped by dataset.

The header of the Genetic Scoring files are slightly different from the one in PGS Catalog (highlighted with '#' below):

```
# OmicsPred ID = OPGS identifier, e.g. 'OPGS000001'
# PGS Name = Genetic Score name, e.g. 'OID21313'
# Trait type = omics type (e.g. proteomics)
# Measurement tissue = tissue name, e.g. venous blood
(UBERON_0013756)
# Measurement platform = platform, e.g. Olink
# Reported Trait = molecular trait, e.g. 'Dickkopf-like protein 1'
# Original Genome Build = Genome build/assembly, e.g. 'GRCh38'
# Number of Variants = Number of variants listed in the Genetic
Score
# Note = extra information, e.g. 'Model extracted as-is from
PredictDB.org'
# Citation = Information about the publication
# License = License and terms of the Genetic Score
use/distribution
rsID  chr_name  chr_position  effect_allele  reference_allele
```

```
effect_weight
…
```

The columns defining the data format itself are listed below:

Column Header	Field Name	Field Description	Is Mandatory?
rsID	dbSNP Accession ID (rsID)	Unique identifier of the variant in dbSNP	**Yes** - Each Genetic Score file must have either an *rsID* column or both a *chr_name* and *chr_position* column to identify the variant.
chr_name	Location - Chromosome	Chromosome associated with the variant	
chr_position	Location - Base pair position within the Chromosome	Chromosomal position associated with the variant	
effect_allele	Effect allele	The allele that's dosage is counted (e.g. {0, 1, 2}) and multiplied by the variant's weight (*effect_weight*) when calculating score. The effect allele is also known as the 'risk allele'	**Yes**
other_allele	Other allele	The other allele(s) at the loci.	**Suggested** - most software requires this for the calculation of scores and matching of the variants to existing genotype data
effect_weight	Variant weight	Value of the effect that is multiplied by the dosage of the effect allele (*effect_allele*) when calculating the score	**Yes**

This table describes the data items that can be captured for each of the data objects in OmicsPred

OmicsPred Data Objects	Data Items	Description	Comments
**Publication** (Identified by OPP ID)	OmicsPred Publication ID (OPP)	Unique identifier created for the publication entries in OmicsPred.	This information is extracted and annotated according to EuropePMC [3].
	PubMed ID (PMID)	PubMed Identification number.	
	Digital Object Identifier (doi)	The doi of the publication.	
	Title	Title of the publication.	
	Author(s)	List of publication authors, the first author is also extracted for a shorter display.	
	Journal	The name of the publication source.	
	Publication Date	Date of publication (with respect to the PMID or doi).	
**Platform**	Platform name	Name of the platform.	
	Platform full name	Full name of the platform.	
	Technic	Short description of the technic used on the platform.	
	Type	Omics type detected by the platform.	
	Version	Platform version (if available).	
**Tissue**	Identifier	External identifier from an ontology (e.g. Uberon)	This information is extracted and annotated according to Experimental Factor Ontology (EFO)[4] using the Ontology Lookup Service (OLS)[5].
	Label	Tissue short name/label	
	Description	Detailed description of the Tissue	
	URL	External URL to the ontology entry	
**Pathway**	External Identifier	Pathway external identifier (e.g. R-HSA-156582 from Reactome)	Linked to Reactome [6]

	External Source	Pathway external source (e.g. Reactome)	
	Parent External Identifier	List of parent Pathway external ID(s)	
	Top Level	Flag to indicate if the Pathway is top-level or not in Reactome	
	Super Pathways	List of top-level parent Pathway	
**Molecular Trait** (Gene, Protein, Metabolite)	Name	Name of the Molecular Trait.	
	External Identifier	Molecular Trait external identifier (e.g. P16581 from UniProt).	Linked to resources such as Ensembl [7], UniProt [8] and ChEBI [9]
	External Source	Molecular Trait external source (e.g. Ensembl, UniProt, ChEBI).	
	Synonyms	List of Molecular Trait synonyms.	
	External References	List of Molecular Trait external references.	
	Biotype	Molecular trait biotype.	Only for Gene
	Retired gene model	Indicate if the Molecular Trait entry has been retired/removed for the external source.	Only for Gene
	Gene	Associated Gene.	Only for Protein
	Pathways	Associated Pathways.	Using mapping provided by Reactome [6]
**Dataset** (Identified by OPD ID)	OmicsPred Dataset ID (OPD)	Unique identifier created for the dataset entries in OmicsPred.	
	Dataset Name	Name of the dataset (if available).	
	Publication	Associated Publication.	
	Platform	Associated Platform.	
	Omics Type	Data type detected by the platform (e.g. gene, protein, metabolite).	
	Genetic Score Development Method	The name or description of the method or computational	

		algorithm used to develop the Genetic Scores of this dataset.	
	Genetic Score Count	Number of Genetic Scores associated with the dataset	
	Tissue	Biological tissue collected to be analyzed on the platform	
	Training Sample(s)	Set of Samples used to create and train the Genetic Scores in the dataset	
	Validation Sample(s)	Set of Samples used to validate the Genetic Scores in the dataset	
	Data files URLs	JSON structure listing the URLs of the different types of datafiles available for download.	
	License/Terms of Use	License/Terms of Use that applies to the Genetic Scores of the Dataset.	
**Genetic Score** (Identified by OPGS ID)	OmicsPred ID	Unique identifier created for the genetic score in OmicsPred.	
	OmicsPred name	Name used by the author to refer to the genetic score before an OmicsPred identifier has been assigned.	
	Reported Molecular Trait	Molecular trait (Gene, Protein, Metabolite, ...) identifier and/or name as reported by the author.	
	Original Genome Build	The version of the genome that the variants present in the Genetic Score are associated with.	
	Number of Variants	Number of variants used to calculate the Genetic Score. In the future this will include a more detailed description of the types of variants present.	
	Genetic Score Development Method	The name or description of the method or computational algorithm used to develop the Genetic Score.	
	Molecular Traits	Molecular Traits linked to the Genetic Score: Gene, Transcript, Protein, Metabolite.	Linked to resources such as Ensembl [7], UniProt [8] and ChEBI [9]

	Ancestry distribution	Distribution of the ancestries in the training and validation of the Genetic Score.	
	Dataset	Associated Dataset	
	License/Terms of Use	License/Terms of Use that applies to the Genetic Score.	
**Cohort**	Cohort name	Cohort short name.	
	Cohort full name	Full name of the cohort.	
	URL	Link to the cohort/study website.	
**Sample**	Sample Number	Number of individuals included in the sample.	
	Sample Cases	Number of individual cases in the sample.	
	Sample Controls	Number of individual controls in the sample.	
	Sample Male Percent	Percentage of male individuals in the sample	
	Broad Ancestry Category	Author reported ancestry is mapped to the best matching ancestry category from the NHGRI-EBI GWAS Catalog framework (Table)[10].	
	Cohort(s)	List of Cohorts used to create the Sample.	
**Performance Metrics** (used to describe training and testing performance for scores)	Genetic Score	Associated Genetic Score	
	Dataset	Associated Dataset (includes Publication, Platform and Tissue)	
	Sample	Associated Sample	
	Cohort label	Shortcut to retrieve the cohort short name/label	
	Metric value	A list of metrics used to evaluate the performance of the Genetic Score within a Sample/Cohort<br>• **Name**: Name of the metric method (e.g. Proportion of the variance explained).	

		• **Name short**: Shorter name of the metric method (e.g. R2).<br>• **Type**: Type of metric (e.g. Pearson's correlation).<br>• **Estimate value**: Metric estimate value.<br>• **P-value**: P-value associated with the Metric.	
**Phenotype** (used to organise PheWAS data)	Identifier	External identifier of the Phenotype.	This information is extracted and annotated according to Experimental Factor Ontology (EFO)[4] using the Ontology Lookup Service (OLS)[5].
	Name	Phenotype name/label.	
	Description	Detailed description of the Phenotype	
	Category	Phenotype category.	
	Source	External source of the Phenotype (e.g. EFO).	
	Mapped Phecode	List of mapped PheCode IDs	
**PheWAS Results**	Score	Associated Score	
	Phenotypes	Associated Phenotypes	
	Sample	Associated Sample (control/cases, percentage male participants).	
	Dataset	Associated Dataset	
	Study type	Type of study (imputed or summary)	
	Trait reported	Reported phenotype	
	Ancestry distribution	Distribution of the ancestries in the Score PheWAS.	
	FDR	False Discovery Rate-adjusted P-value (<0.5).	
	Other data	• R^2<br>• HR: Hazard Ratio with confidence interval<br>• Z-score: Standard score	

		• P-value<br>• Bonferroni<br>• Effect size<br>• Var_gene_exp: Gene expression variance	
	Variants number used	Number of variants from the genetic score used in the PheWAS	
	Variants fraction found	Fraction of variants used from the genetic score used in the PheWAS	

2. OmicsPred data access and implementation

Data in OmicsPred can be currently accessed in the following ways:

- **Downloads** from OmicsPred are provisioned by the Box™ service. All the available downloads are listed on the webpage https://www.omicspred.org/downloads. It includes several types of downloads, grouped by dataset:
    - **Metadata files** (excel spreadsheets) describing all genetic scores in terms of their publication source, detection platform, samples, tissues, phenotypes, molecular traits and related performance metrics.
    - **Genetic Scores** are provided as:
        - **Scoring files** compatible with `pgsc_calc`[2] (https://pgsc-calc.readthedocs.io/en/latest/). The data format is described above and on the PGS Catalog website (https://www.pgscatalog.org/downloads/#dl_ftp_scoring).
        - **Databases in PredictDB format** (https://predictdb.org/) that are compatible with MetaXcan tools[11]. Associated covariance files are also available.
- A **REST API** is also provided to allow programmatic access and querying of OmicsPred metadata, better enabling other applications to be built on top of the resource. Endpoints to retrieve all or individual OmicsPred data objects (Publications, Datasets, Genetic Scores, Platforms, Samples, Molecular Traits, Performance Metrics, PheWAS) are available (details at: https://rest.omicspred.org/).

OmicsPred is also indexed on FAIRsharing.org (doi:10.25504/FAIRsharing.784c89).

Additional bibliographic information for OmicsPred **Publication** objects are retrieved from Europe PMC[3] (e.g. title, authors, journal, publication dates). Additional information for each ontology term (e.g. for the Tissues and Phenotypes objects) from the EFO[4] are obtained using the EMBL-EBI Ontology Lookup Service (OLS)[5].

The OmicsPred website is developed using ReactJS (Vite version 8; https://vite.dev/) and is fed by a private REST API. The two REST APIs (public and private) and the database are developed using the Django framework (version 5.2; https://djangoproject.com) in Python

(version 3.13; https://www.python.org) with a PostgreSQL database (version 15; https://www.postgresql.org/). The search functionality is built using ElasticSearch (v7.17; https://www.elastic.co).The website, database, REST API and search index are all deployed on the Google Cloud platform (https://cloud.google.com/). The codebase for OmicsPred can be viewed within our public GitHub repository (https://github.com/OmicsPred), currently provided under an Apache 2.0 License.

3. Inclusion Criteria for OmicsPred

For a publication's data to be included in OmicsPred it must contain one of the following:

- **Newly developed genetic scores.** This includes the following information about the score and its predictive ability (evaluated on samples not used to develop the score):
    - Variant information necessary to apply the score to new samples (variant rsID and/or genomic position, weights/effect sizes, effect allele, genome build).
        - *Optional*: covariance matrix for variants to support summary-statistics based analyses (e.g. MetaXcan).
    - Information about score development (computational method, variant selection, relevant parameters).
    - Descriptions of the samples used to develop and evaluate each score .
    - Establishment of analytic validity and a description of each score's predictive performance, typically the proportion of the variance explained (R^2).
- **An evaluation of a previously developed OmicsPred score or dataset**, for instance on samples not used for development. The requirements for description would be the same as for the evaluation of a new genetic score.
- **PheWAS results of genetic scores** for organismal phenotypes (e.g. disease traits) can also be accepted, provided submitters can demonstrate impact to downstream users.

A complete description of the metadata captured is described in Supplemental Note 2 and the most up to date version can be found on the website(https://www.omicspred.org/docs). To ensure comprehensiveness of the resource we will actively identify new publications with scores via literature search and contact authors directly to encourage submission to the resource.

Supplemental Tables

Supplementary Table 1. Summary of MVP PheWAS analyses and results. Eight OmicsPred score datasets derived from blood and plasma were evaluated. Association analyses were performed using the S-PrediXcan function implemented in MetaXcan, with MVP GWAS summary statistics for PheCodes traits as input. Each score dataset was analysed only in GWAS with ancestry similar to the training ancestry. The resulting p-values were corrected for multiple testing within each PheCode-dataset combination. Scores with less than 75% of variant match rate to the GWAS summary stats were excluded from analysis.

Dataset ID	Tissue	Publication	Omic Layer	Platform	Training Cohort (Ancestry)	#Scores	#GWAS Traits	GWAS Ancestry	FDR Significant Associations
OPD000001	blood plasma	OPP000001	Proteomics	Somalogic	INTERVAL (European)	2,384	1233	European	26,109
OPD000005	blood	OPP000001	Transcripo-mics	RNAseq - Expression	INTERVAL (European)	13,668	1233	European	39,586
OPD000006	blood plasma	OPP000002	Transcripo-mics	Olink	UK Biobank (European)	2,612	1233	European	53,669
OPD000056	venous blood	OPP000003	Transcripo-mics	RNAseq - Expression	GTEx (Largely European)	7,252	1233	European	30,552
OPD000105	venous blood	OPP000003	Transcripo-mics	RNAseq - Splicing	GTEx (Largely European)	8,724	1233	European	36,442
OPD000205	blood plasma	OPP000004	Proteomics	Somalogic	TOPMed - MESA (African)	557	986	African	275
OPD000213	blood plasma	OPP000004	Proteomics	Somalogic	TOPMed - MESA (Ad Mixed American)	584	719	Admixed American	117
OPD000214	blood plasma	OPP000006	Proteomics	Somalogic	ARIC (African)	1359	986	African	419
OPD000215	blood plasma	OPP000006	Proteomics	Somalogic	ARIC (European)	1310	1233	European	6969

Supplemental Figures

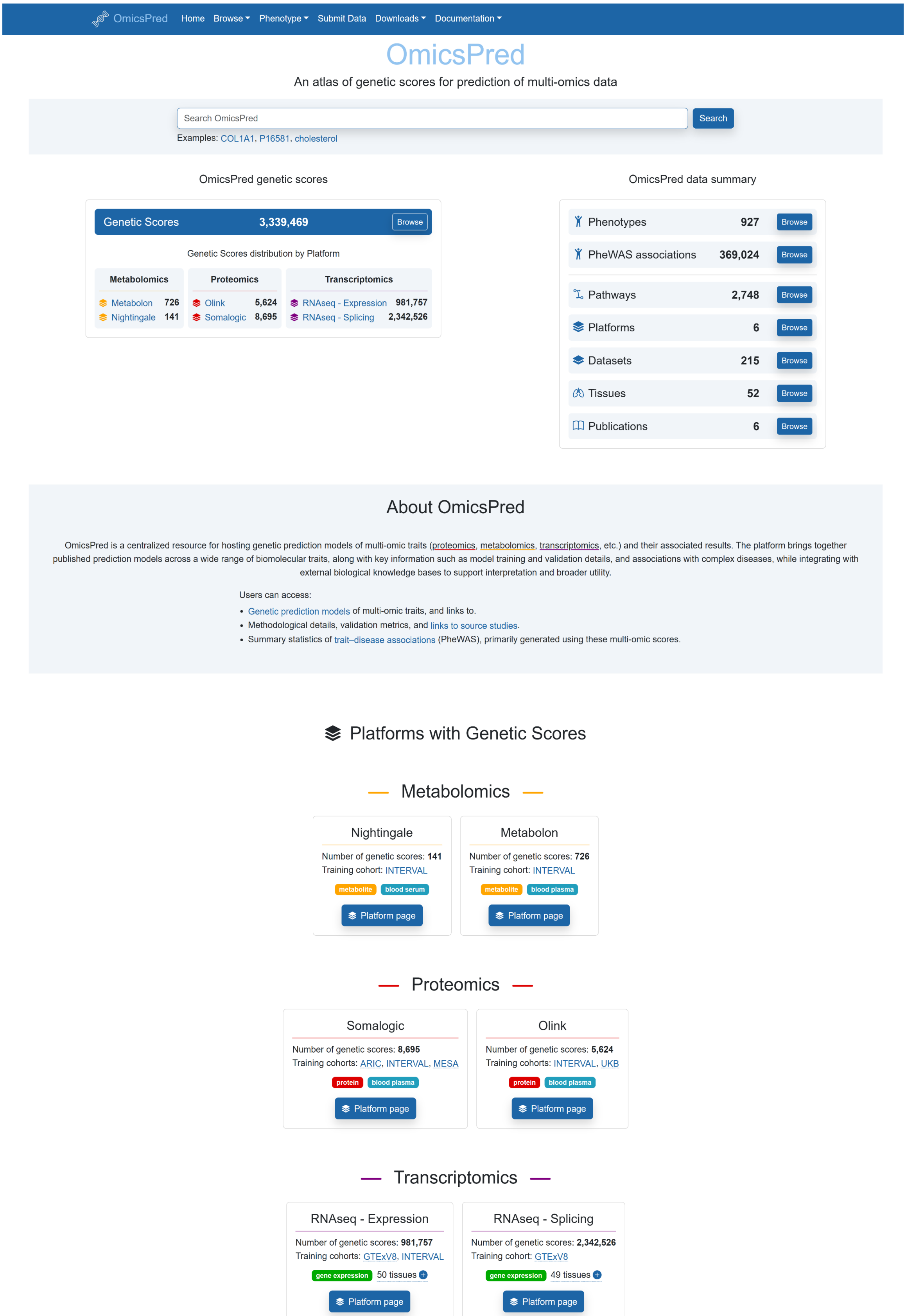

Figure S1. Screenshot of the OmicsPred landing page (www.omicspred.org). This page gives users a visual overview of the data indexed in the Catalog.

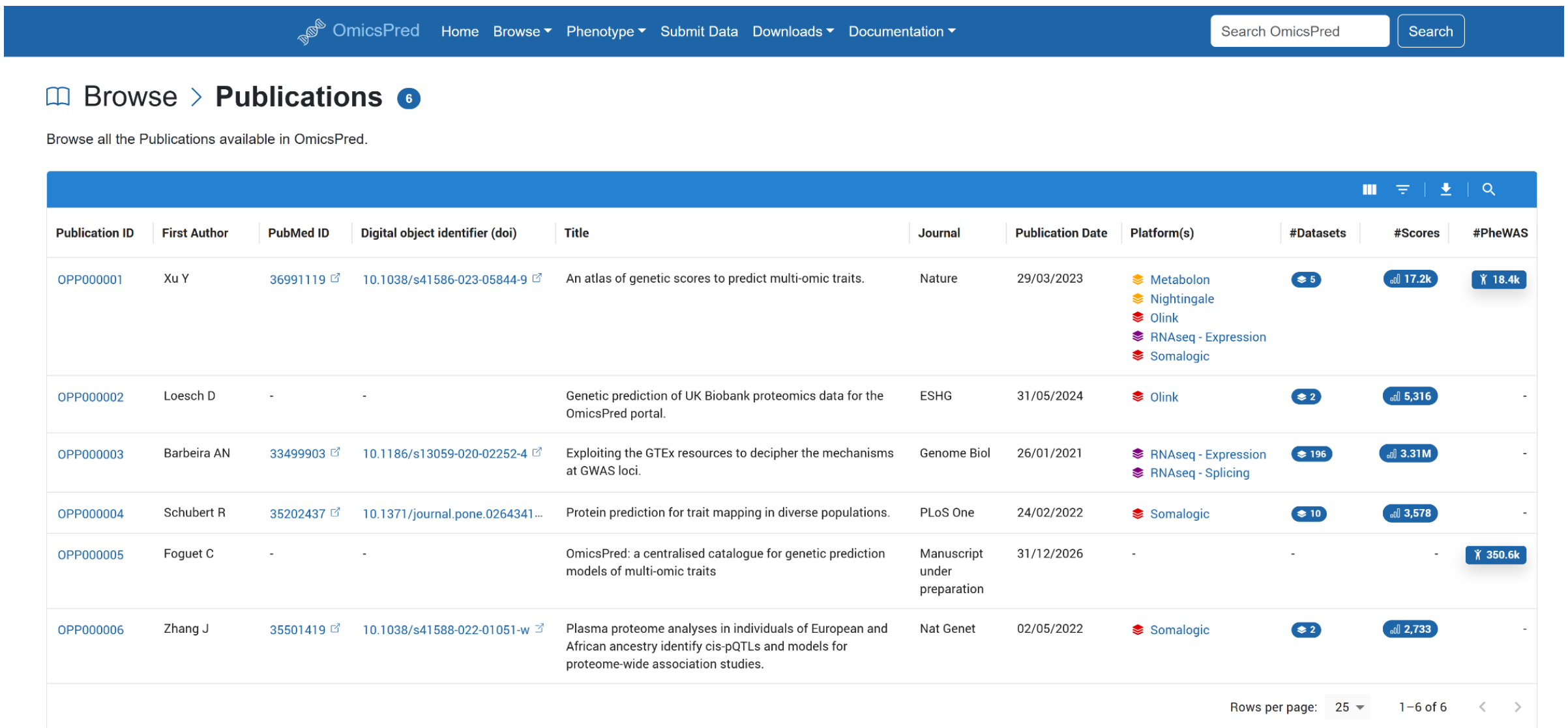

Figure S2. Screenshot of the OmicsPred publication page (https://www.omicspred.org/publications). This page allows users to browse the list of publications included in the catalog.

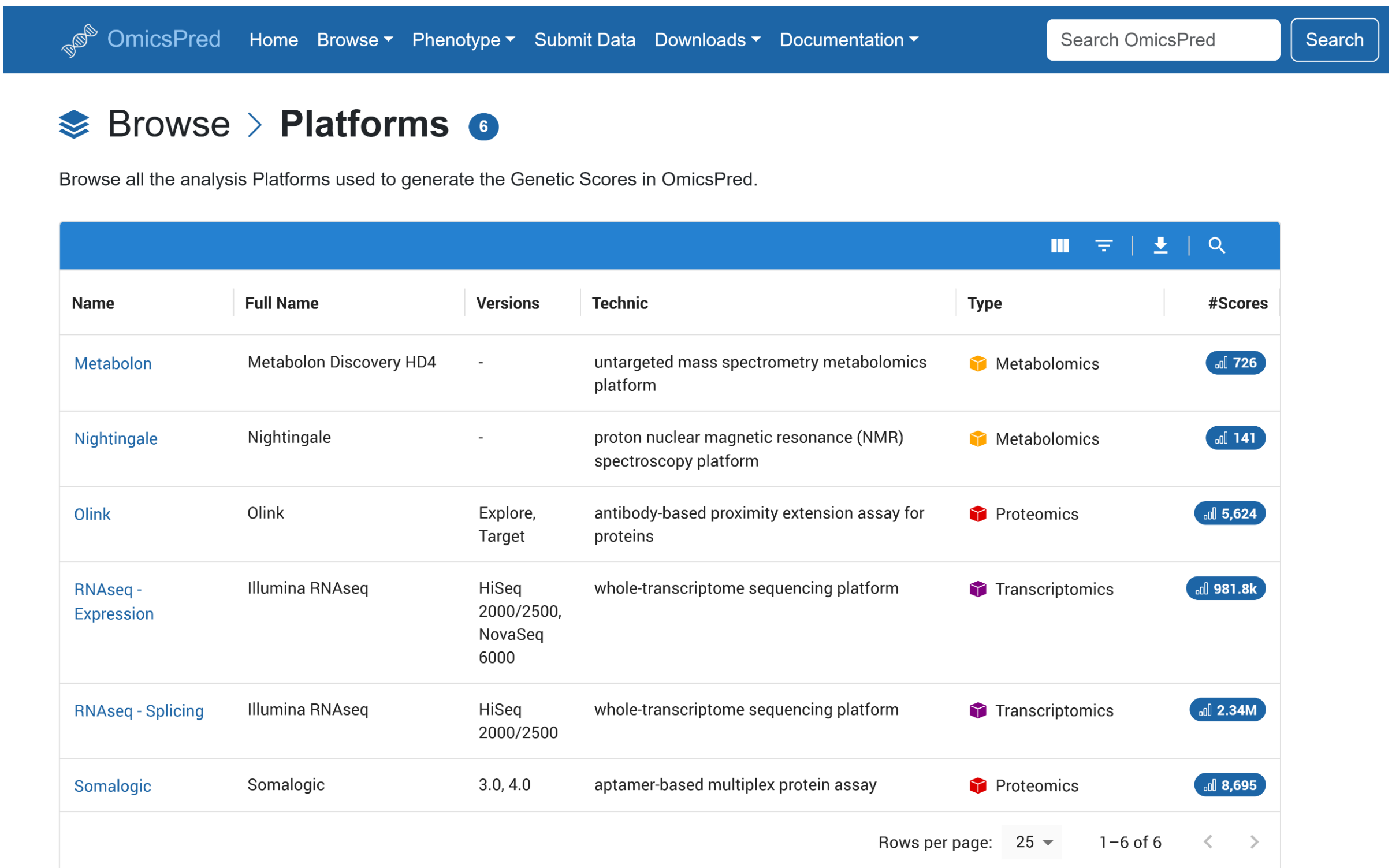

Figure S3. Screenshot of the OmicsPred platforms page (https://www.omicspred.org/platforms). This page allows users to browse the list of omics platforms included in the catalog.

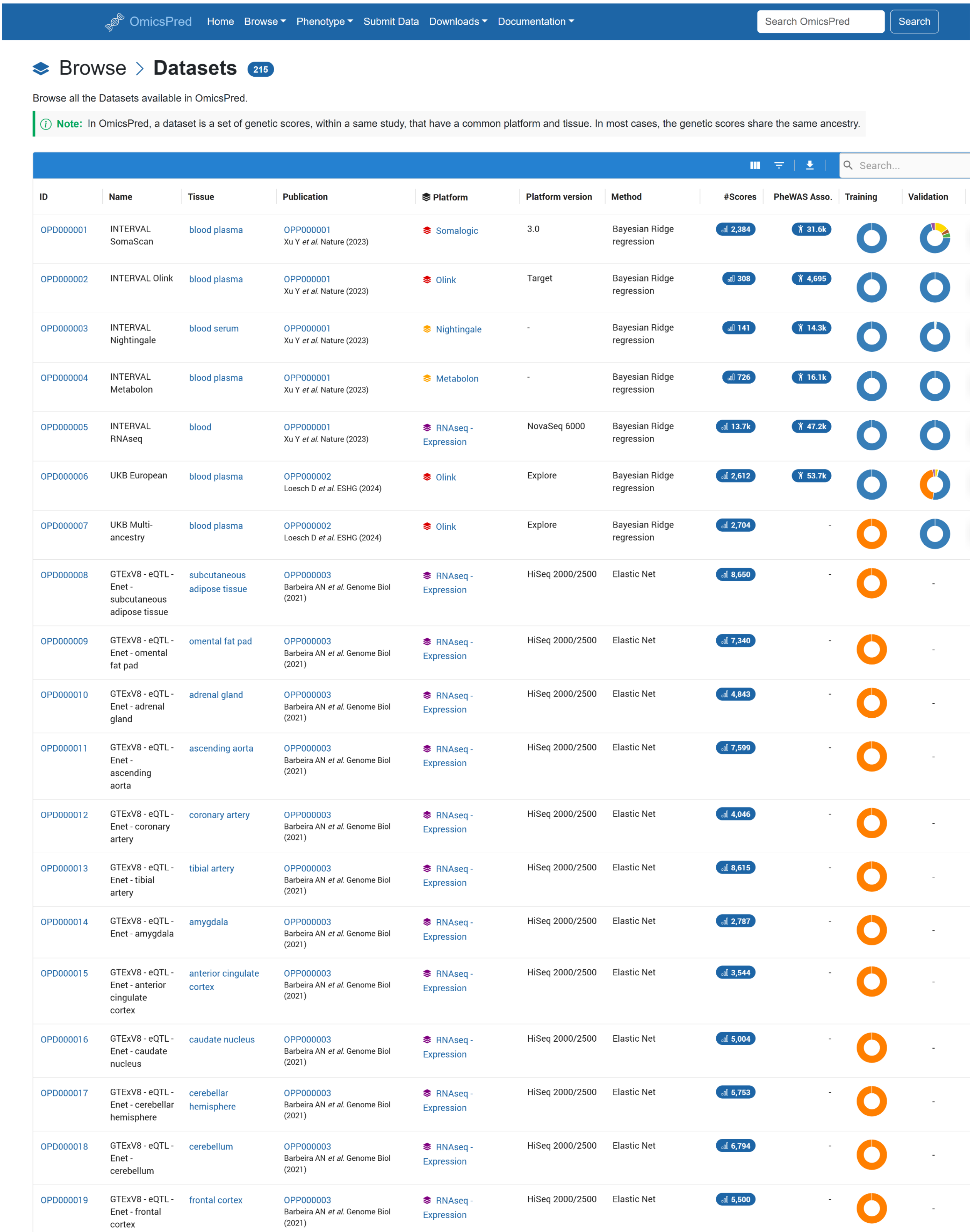

OmicsPred | Home | Browse | Phenotype | Submit Data | Downloads | Documentation | Search OmicsPred | Search

Browse > Datasets 215

Browse all the Datasets available in OmicsPred.

Note: In OmicsPred, a dataset is a set of genetic scores, within a same study, that have a common platform and tissue. In most cases, the genetic scores share the same ancestry.

ID	Name	Tissue	Publication	Platform	Platform version	Method	#Scores	PheWAS Asso.	Training	Validation
OPD000001	INTERVAL SomaScan	blood plasma	OPP000001 Xu Y *et al.* Nature (2023)	Somalogic	3.0	Bayesian Ridge regression	2,384	31.6k		
OPD000002	INTERVAL Olink	blood plasma	OPP000001 Xu Y *et al.* Nature (2023)	Olink	Target	Bayesian Ridge regression	308	4,695		
OPD000003	INTERVAL Nightingale	blood serum	OPP000001 Xu Y *et al.* Nature (2023)	Nightingale	-	Bayesian Ridge regression	141	14.3k		
OPD000004	INTERVAL Metabolon	blood plasma	OPP000001 Xu Y *et al.* Nature (2023)	Metabolon	-	Bayesian Ridge regression	726	16.1k		
OPD000005	INTERVAL RNAseq	blood	OPP000001 Xu Y *et al.* Nature (2023)	RNAseq - Expression	NovaSeq 6000	Bayesian Ridge regression	13.7k	47.2k		
OPD000006	UKB European	blood plasma	OPP000002 Loesch D *et al.* ESHG (2024)	Olink	Explore	Bayesian Ridge regression	2,612	53.7k		
OPD000007	UKB Multi-ancestry	blood plasma	OPP000002 Loesch D *et al.* ESHG (2024)	Olink	Explore	Bayesian Ridge regression	2,704	-		
OPD000008	GTExV8 - eQTL - Enet - subcutaneous adipose tissue	subcutaneous adipose tissue	OPP000003 Barbeira AN *et al.* Genome Biol (2021)	RNAseq - Expression	HiSeq 2000/2500	Elastic Net	8,650	-		-
OPD000009	GTExV8 - eQTL - Enet - omental fat pad	omental fat pad	OPP000003 Barbeira AN *et al.* Genome Biol (2021)	RNAseq - Expression	HiSeq 2000/2500	Elastic Net	7,340	-		-
OPD000010	GTExV8 - eQTL - Enet - adrenal gland	adrenal gland	OPP000003 Barbeira AN *et al.* Genome Biol (2021)	RNAseq - Expression	HiSeq 2000/2500	Elastic Net	4,843	-		-
OPD000011	GTExV8 - eQTL - Enet - ascending aorta	ascending aorta	OPP000003 Barbeira AN *et al.* Genome Biol (2021)	RNAseq - Expression	HiSeq 2000/2500	Elastic Net	7,599	-		-
OPD000012	GTExV8 - eQTL - Enet - coronary artery	coronary artery	OPP000003 Barbeira AN *et al.* Genome Biol (2021)	RNAseq - Expression	HiSeq 2000/2500	Elastic Net	4,046	-		-
OPD000013	GTExV8 - eQTL - Enet - tibial artery	tibial artery	OPP000003 Barbeira AN *et al.* Genome Biol (2021)	RNAseq - Expression	HiSeq 2000/2500	Elastic Net	8,615	-		-
OPD000014	GTExV8 - eQTL - Enet - amygdala	amygdala	OPP000003 Barbeira AN *et al.* Genome Biol (2021)	RNAseq - Expression	HiSeq 2000/2500	Elastic Net	2,787	-		-
OPD000015	GTExV8 - eQTL - Enet - anterior cingulate cortex	anterior cingulate cortex	OPP000003 Barbeira AN *et al.* Genome Biol (2021)	RNAseq - Expression	HiSeq 2000/2500	Elastic Net	3,544	-		-
OPD000016	GTExV8 - eQTL - Enet - caudate nucleus	caudate nucleus	OPP000003 Barbeira AN *et al.* Genome Biol (2021)	RNAseq - Expression	HiSeq 2000/2500	Elastic Net	5,004	-		-
OPD000017	GTExV8 - eQTL - Enet - cerebellar hemisphere	cerebellar hemisphere	OPP000003 Barbeira AN *et al.* Genome Biol (2021)	RNAseq - Expression	HiSeq 2000/2500	Elastic Net	5,753	-		-
OPD000018	GTExV8 - eQTL - Enet - cerebellum	cerebellum	OPP000003 Barbeira AN *et al.* Genome Biol (2021)	RNAseq - Expression	HiSeq 2000/2500	Elastic Net	6,794	-		-
OPD000019	GTExV8 - eQTL - Enet - frontal cortex	frontal cortex	OPP000003 Barbeira AN *et al.* Genome Biol (2021)	RNAseq - Expression	HiSeq 2000/2500	Elastic Net	5,500	-		-

Figure S4. Screenshot of the OmicsPred datasets page (https://www.omicspred.org/datasets). This page allows users to browse the list of datasets (groups of scores derived from a single publication that share a common platform, tissue, and training methodology) included in the catalog.

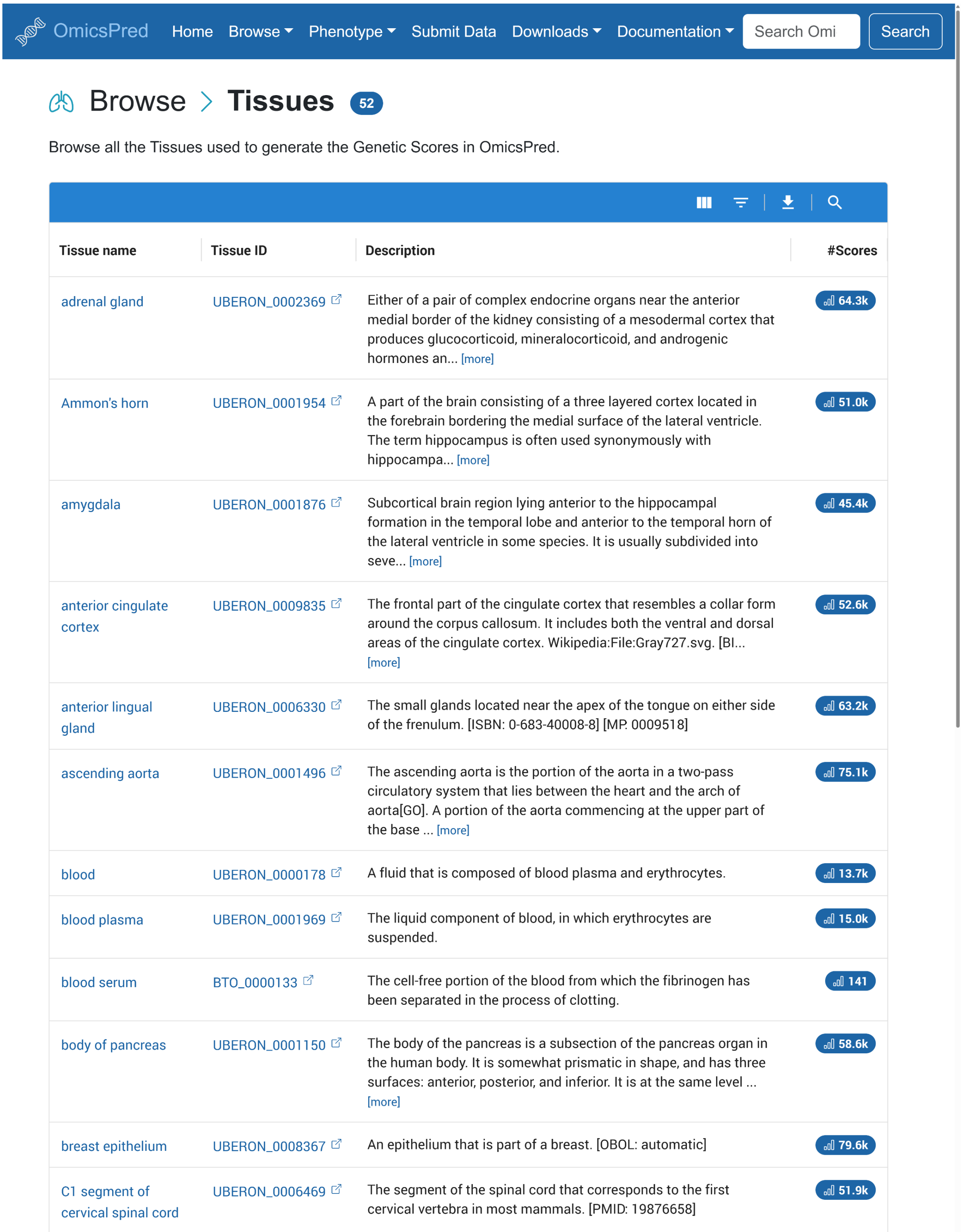

OmicsPred Home Browse Phenotype Submit Data Downloads Documentation Search Omi Search

Browse > Tissues 52

Browse all the Tissues used to generate the Genetic Scores in OmicsPred.

Tissue name	Tissue ID	Description	#Scores
adrenal gland	UBERON_0002369	Either of a pair of complex endocrine organs near the anterior medial border of the kidney consisting of a mesodermal cortex that produces glucocorticoid, mineralocorticoid, and androgenic hormones an... [more]	64.3k
Ammon's horn	UBERON_0001954	A part of the brain consisting of a three layered cortex located in the forebrain bordering the medial surface of the lateral ventricle. The term hippocampus is often used synonymously with hippocampa... [more]	51.0k
amygdala	UBERON_0001876	Subcortical brain region lying anterior to the hippocampal formation in the temporal lobe and anterior to the temporal horn of the lateral ventricle in some species. It is usually subdivided into seve... [more]	45.4k
anterior cingulate cortex	UBERON_0009835	The frontal part of the cingulate cortex that resembles a collar form around the corpus callosum. It includes both the ventral and dorsal areas of the cingulate cortex. Wikipedia:File:Gray727.svg. [Bl... [more]	52.6k
anterior lingual gland	UBERON_0006330	The small glands located near the apex of the tongue on either side of the frenulum. [ISBN: 0-683-40008-8] [MP: 0009518]	63.2k
ascending aorta	UBERON_0001496	The ascending aorta is the portion of the aorta in a two-pass circulatory system that lies between the heart and the arch of aorta[GO]. A portion of the aorta commencing at the upper part of the base ... [more]	75.1k
blood	UBERON_0000178	A fluid that is composed of blood plasma and erythrocytes.	13.7k
blood plasma	UBERON_0001969	The liquid component of blood, in which erythrocytes are suspended.	15.0k
blood serum	BTO_0000133	The cell-free portion of the blood from which the fibrinogen has been separated in the process of clotting.	141
body of pancreas	UBERON_0001150	The body of the pancreas is a subsection of the pancreas organ in the human body. It is somewhat prismatic in shape, and has three surfaces: anterior, posterior, and inferior. It is at the same level ... [more]	58.6k
breast epithelium	UBERON_0008367	An epithelium that is part of a breast. [OBOL: automatic]	79.6k
C1 segment of cervical spinal cord	UBERON_0006469	The segment of the spinal cord that corresponds to the first cervical vertebra in most mammals. [PMID: 19876658]	51.9k

Figure S5. Screenshot of the OmicsPred Tissues page (https://www.omicspred.org/tissues). This page allows users to browse the list of tissues included in the catalog, important for navigating to individual cell/tissue-type pages necessary for identifying context-specific genetic predictors.

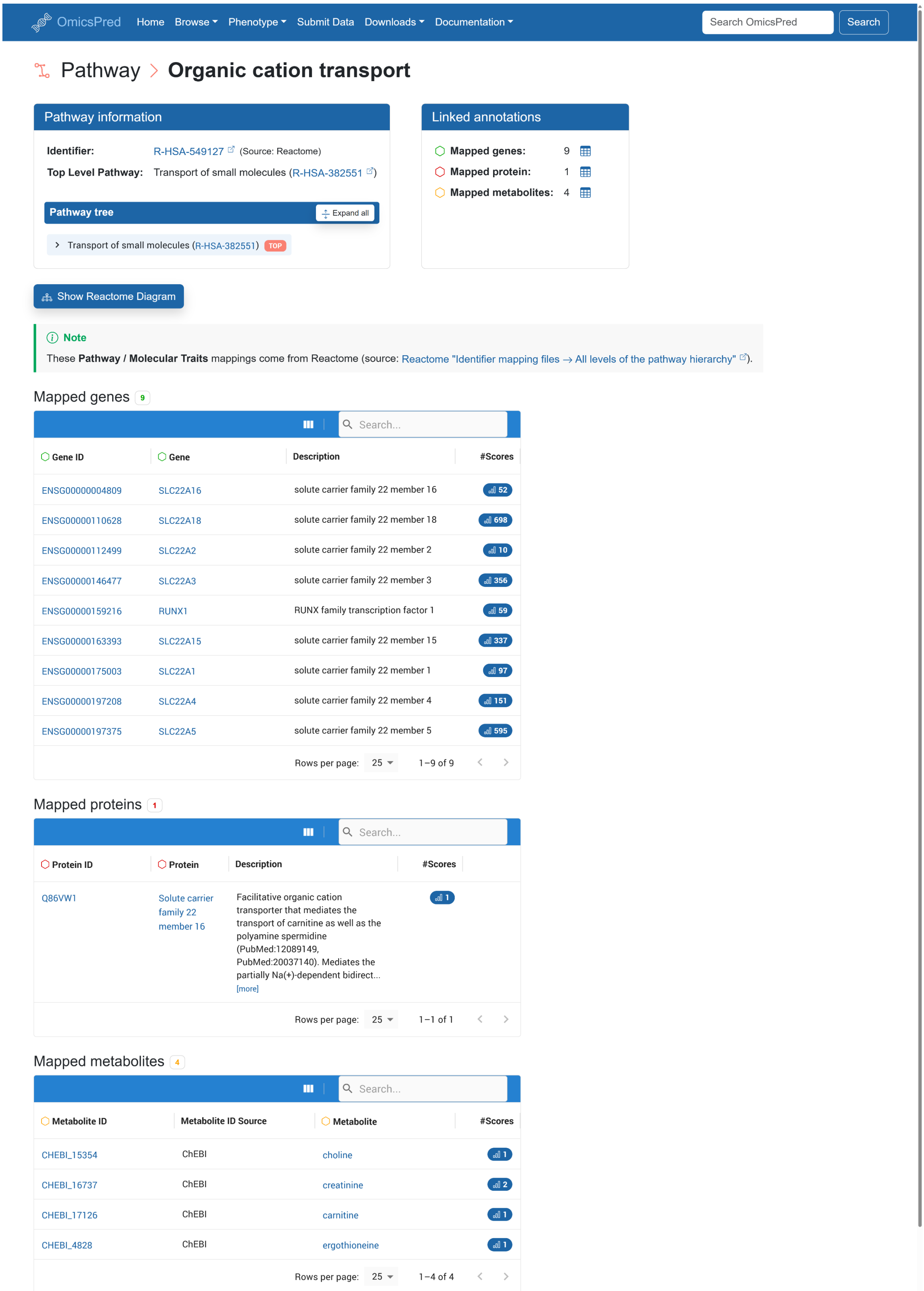

Figure S5. Screenshot of an OmicsPred Pathway page (https://www.omicspred.org/pathway/R-HSA-549127). Example of an OmicsPred pathway page displaying the scores that are mapped to a specific Reactome pathway (R-HSA-549127 in this example). Pathway-level mapping provides key biological context, facilitating functional interpretation and integration between different datasets.

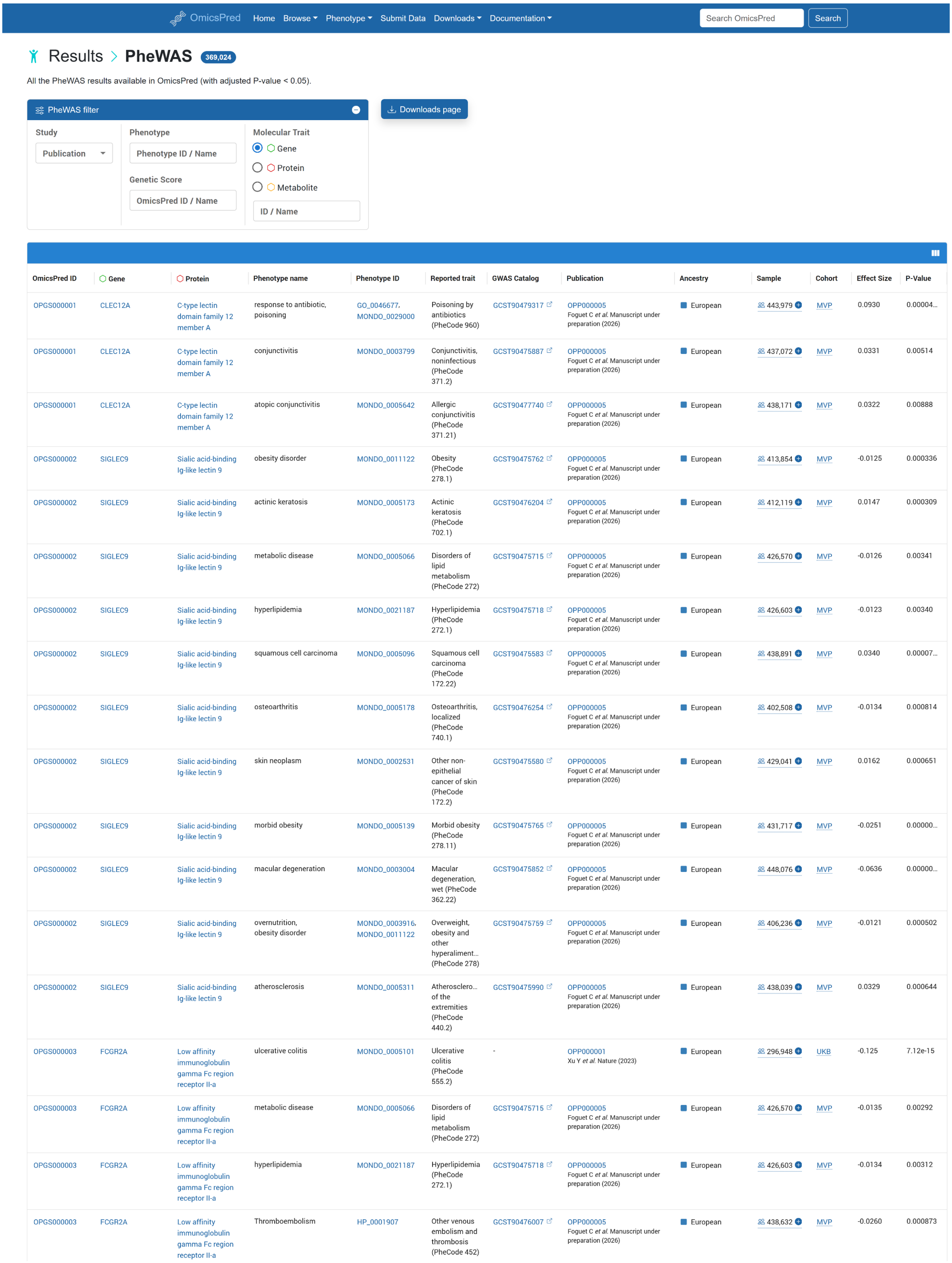

OmicsPred ID	Gene	Protein	Phenotype name	Phenotype ID	Reported trait	GWAS Catalog	Publication	Ancestry	Sample	Cohort	Effect Size	P-Value
OPGS000001	CLEC12A	C-type lectin domain family 12 member A	response to antibiotic, poisoning	GO_0046677, MONDO_0029000	Poisoning by antibiotics (PheCode 960)	GCST90479317	OPP000005 Foguet C *et al.* Manuscript under preparation (2026)	European	443,979	MVP	0.0930	0.00004...
OPGS000001	CLEC12A	C-type lectin domain family 12 member A	conjunctivitis	MONDO_0003799	Conjunctivitis, noninfectious (PheCode 371.2)	GCST90475887	OPP000005 Foguet C *et al.* Manuscript under preparation (2026)	European	437,072	MVP	0.0331	0.00514
OPGS000001	CLEC12A	C-type lectin domain family 12 member A	atopic conjunctivitis	MONDO_0005642	Allergic conjunctivitis (PheCode 371.21)	GCST90477740	OPP000005 Foguet C *et al.* Manuscript under preparation (2026)	European	438,171	MVP	0.0322	0.00888
OPGS000002	SIGLEC9	Sialic acid-binding Ig-like lectin 9	obesity disorder	MONDO_0011122	Obesity (PheCode 278.1)	GCST90475762	OPP000005 Foguet C *et al.* Manuscript under preparation (2026)	European	413,854	MVP	-0.0125	0.000336
OPGS000002	SIGLEC9	Sialic acid-binding Ig-like lectin 9	actinic keratosis	MONDO_0005173	Actinic keratosis (PheCode 702.1)	GCST90476204	OPP000005 Foguet C *et al.* Manuscript under preparation (2026)	European	412,119	MVP	0.0147	0.000309
OPGS000002	SIGLEC9	Sialic acid-binding Ig-like lectin 9	metabolic disease	MONDO_0005066	Disorders of lipid metabolism (PheCode 272)	GCST90475715	OPP000005 Foguet C *et al.* Manuscript under preparation (2026)	European	426,570	MVP	-0.0126	0.00341
OPGS000002	SIGLEC9	Sialic acid-binding Ig-like lectin 9	hyperlipidemia	MONDO_0021187	Hyperlipidemia (PheCode 272.1)	GCST90475718	OPP000005 Foguet C *et al.* Manuscript under preparation (2026)	European	426,603	MVP	-0.0123	0.00340
OPGS000002	SIGLEC9	Sialic acid-binding Ig-like lectin 9	squamous cell carcinoma	MONDO_0005096	Squamous cell carcinoma (PheCode 172.22)	GCST90475583	OPP000005 Foguet C *et al.* Manuscript under preparation (2026)	European	438,891	MVP	0.0340	0.00007...
OPGS000002	SIGLEC9	Sialic acid-binding Ig-like lectin 9	osteoarthritis	MONDO_0005178	Osteoarthritis, localized (PheCode 740.1)	GCST90476254	OPP000005 Foguet C *et al.* Manuscript under preparation (2026)	European	402,508	MVP	-0.0134	0.000814
OPGS000002	SIGLEC9	Sialic acid-binding Ig-like lectin 9	skin neoplasm	MONDO_0002531	Other non-epithelial cancer of skin (PheCode 172.2)	GCST90475580	OPP000005 Foguet C *et al.* Manuscript under preparation (2026)	European	429,041	MVP	0.0162	0.000651
OPGS000002	SIGLEC9	Sialic acid-binding Ig-like lectin 9	morbid obesity	MONDO_0005139	Morbid obesity (PheCode 278.11)	GCST90475765	OPP000005 Foguet C *et al.* Manuscript under preparation (2026)	European	431,717	MVP	-0.0251	0.00000...
OPGS000002	SIGLEC9	Sialic acid-binding Ig-like lectin 9	macular degeneration	MONDO_0003004	Macular degeneration, wet (PheCode 362.22)	GCST90475852	OPP000005 Foguet C *et al.* Manuscript under preparation (2026)	European	448,076	MVP	-0.0636	0.00000...
OPGS000002	SIGLEC9	Sialic acid-binding Ig-like lectin 9	overnutrition, obesity disorder	MONDO_0003916, MONDO_0011122	Overweight, obesity and other hyperaliment... (PheCode 278)	GCST90475759	OPP000005 Foguet C *et al.* Manuscript under preparation (2026)	European	406,236	MVP	-0.0121	0.000502
OPGS000002	SIGLEC9	Sialic acid-binding Ig-like lectin 9	atherosclerosis	MONDO_0005311	Atherosclero... of the extremities (PheCode 440.2)	GCST90475990	OPP000005 Foguet C *et al.* Manuscript under preparation (2026)	European	438,039	MVP	0.0329	0.000644
OPGS000003	FCGR2A	Low affinity immunoglobulin gamma Fc region receptor II-a	ulcerative colitis	MONDO_0005101	Ulcerative colitis (PheCode 555.2)	-	OPP000001 Xu Y *et al.* Nature (2023)	European	296,948	UKB	-0.125	7.12e-15
OPGS000003	FCGR2A	Low affinity immunoglobulin gamma Fc region receptor II-a	metabolic disease	MONDO_0005066	Disorders of lipid metabolism (PheCode 272)	GCST90475715	OPP000005 Foguet C *et al.* Manuscript under preparation (2026)	European	426,570	MVP	-0.0135	0.00292
OPGS000003	FCGR2A	Low affinity immunoglobulin gamma Fc region receptor II-a	hyperlipidemia	MONDO_0021187	Hyperlipidemia (PheCode 272.1)	GCST90475718	OPP000005 Foguet C *et al.* Manuscript under preparation (2026)	European	426,603	MVP	-0.0134	0.00312
OPGS000003	FCGR2A	Low affinity immunoglobulin gamma Fc region receptor II-a	Thromboembolism	HP_0001907	Other venous embolism and thrombosis (PheCode 452)	GCST90476007	OPP000005 Foguet C *et al.* Manuscript under preparation (2026)	European	438,632	MVP	-0.0260	0.000873

Figure S6. Screenshot of the OmicsPred PheWAS page (https://www.omicspred.org/phewas). This page allows users to browse the associations between genetically predicted molecular traits and disease outcomes, useful for hypothesis generation.

Supplemental References

1. Wilkinson, M. D. *et al.* The FAIR Guiding Principles for scientific data management and stewardship. *Sci. Data* **3**, 160018 (2016).

2. Lambert, S. A. *et al.* Enhancing the Polygenic Score Catalog with tools for score calculation and ancestry normalization. *Nat. Genet.* **56**, 1989–1994 (2024).

3. Ferguson, C. *et al.* Europe PMC in 2020. *Nucleic Acids Res.* **49**, D1507–D1514 (2020).

4. Malone, J. *et al.* Modeling sample variables with an Experimental Factor Ontology. https://dx.doi.org/10.1093/bioinformatics/btq099.

5. McLaughlin, J., Lagrimas, J., Iqbal, H., Parkinson, H. & Harmse, H. OLS4: a new Ontology Lookup Service for a growing interdisciplinary knowledge ecosystem. *Bioinformatics* **41**, btaf279 (2025).

6. Ragueneau, E. *et al.* The Reactome Knowledgebase 2026. *Nucleic Acids Res.* **54**, D673–D681 (2026).

7. Dyer, S. C. *et al.* Ensembl 2025. *Nucleic Acids Res.* **53**, D948–D957 (2025).

8. The UniProt Consortium. UniProt: the Universal Protein Knowledgebase in 2025. *Nucleic Acids Res.* **53**, D609–D617 (2025).

9. Malik, A. *et al.* ChEBI: re-engineered for a sustainable future. *Nucleic Acids Res.* **54**, D1768–D1778 (2026).

10. Morales, J. *et al.* A standardized framework for representation of ancestry data in genomics studies, with application to the NHGRI-EBI GWAS Catalog. *Genome Biol.* **19**, 21 (2018).

11. Barbeira, A. N. *et al.* Exploring the phenotypic consequences of tissue specific gene expression variation inferred from GWAS summary statistics. *Nat. Commun.* **9**, 1825 (2018).
